# Out of Sight, Out of Mind: Refugees Are Just the Tip of the Iceberg. An Illustration Using the Cases of Depression and Posttraumatic Stress Disorder

**DOI:** 10.3389/fpsyt.2020.00179

**Published:** 2020-03-06

**Authors:** Nexhmedin Morina, Thole H. Hoppen, Stefan Priebe

**Affiliations:** ^1^Institute of Psychology, University of Münster, Münster, Germany; ^2^Unit for Social and Community Psychiatry (WHO Collaborating Centre for Mental Health Services Development), Queen Mary University of London, London, United Kingdom

**Keywords:** war, mass conflict, refugees, PTSD, depression

## Introduction

Recent and ongoing mass conflicts have forced millions of individuals to flee to other countries. According to the United Nations High Commissioner for Refugees, about 26 million individuals were living as refugees in 2018, having fled warfare or severe violations of human rights (1). In many countries, the arrival of refugees has led to extensive media attention and public debates about the extent to which refugees should be accepted or rejected in a country. These debates have become a dominating issue in recent political election campaigns, e.g., in Austria, Germany, Hungary, and Italy, with a substantial impact on international relations. Large numbers of refugees can present major challenges to health care systems in host societies, in particular to mental health care (2). Accordingly, prevalences of mental disorders among refugees and different treatments for refugees with mental disorders have been addressed in numerous empirical studies (3). Whilst refugees are a major concern for all the host countries, numerically they represent only a small proportion of all war survivors in the world. On a global level, many more survivors of war do not want or are not able to take refuge, but instead continue to live in the area of the former or ongoing conflict. For these people, there is no specific UN Agency—as there is for refugees—and official statistics do not exist.

## Posttraumatic Stress Disorder (PTSD) and Major Depression

Experiencing war can have detrimental effects for mental health and lead to various forms of severe distress. PTSD and major depression are the most prevalent in survivors of war. In a recent systematic review and meta-analysis of epidemiological surveys that had utilized a random sampling procedure from the general population in war-afflicted regions and applied structured psychiatric interviews, we reported that 24 and 23% of adult war survivors are likely to suffer from PTSD and depression, respectively (4). Furthermore, epidemiological research suggests that about 10% of war survivors suffer from concomitant PTSD and depression, indicating that about 34% of war survivors suffer from at least one of these conditions (4). Two of the most comprehensive systematic reviews and meta-analyses that provided pooled prevalences for PTSD and depression among refugees reported variable findings. Fazel et al. (5) focused on refugees resettled in Western countries and reported that 9 and 5% of them suffer from PTSD and depression, respectively. Steel et al. (6) focused on surveys with refugees and other conflict-affected populations from around the globe and reported that 31% of conflict-affected populations suffer from each disorder. Despite the very variable findings on prevalences of mental disorders in refugees, on average prevalences of PTSD and depression should not be higher than those in populations who stayed in the area of conflict (3).

**Figure 1:**
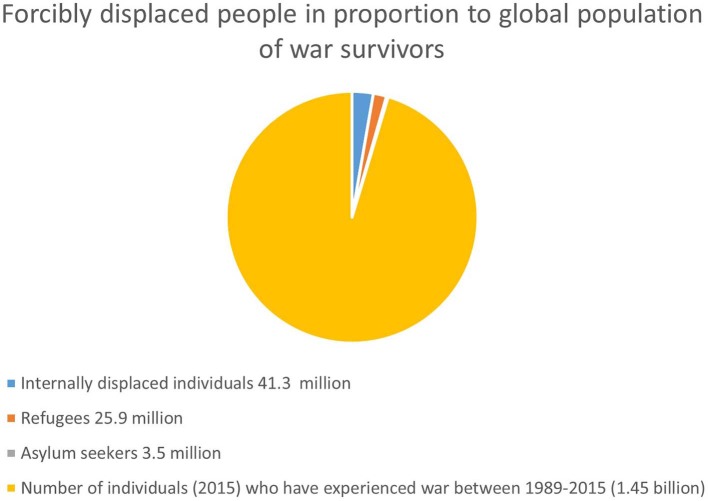
Global population of forcibly displaced individuals in proportion to global population of war survivors.

For an estimate of the total number of war survivors living in the areas of former or ongoing conflict, we used two sources. First, geo-referenced data of the Uppsala Conflict Data Program (UCDP) suggest that between 1989 and 2015 warfare took place in the territories of 47 countries (7). The UCDP defines wars as armed conflicts that result in at least 1,000 battle-related deaths in one calendar year and in which at least one actor is the government of a state. This definition excludes armed civil conflicts where the government is not directly involved. The 47 countries are therefore a conservative figure and a likely underestimation, but still a reasonable guide for our estimate. We then used population estimates from the United Nations (8) to estimate the number of people living in these 47 countries. In 2015, about 1.45 billion individuals lived in these 47 countries, 1.02 billion of them were adults, and 453 million were children and adolescents. This large number contrasts to the nearly 26 million refugees worldwide (see Figure 1).

Assuming that surveys conducted in general population in war-afflicted regions provide accurate estimates of PTSD and depression, their pooled prevalences translate into nearly 240 million adult survivors of war with PTSD and just as many with depression. Furthermore, about 350 million adult war survivors are estimated to suffer from either PTSD or depression, or from both conditions (4). Current surveys might overestimate prevalences of these two conditions due to methodological shortcomings and as time or other factors may contribute to remission over time. However, research indicates that PTSD and depression are rather chronic disorders if untreated and the countries with a recent history of war represent low and middle-income countries (LMICs) with very limited mental health services. Assuming similar prevalences in war surviving refugees and non-refugees (3), the potentially 350 million adult war survivors with PTSD and/or depression who live in areas of conflict contrast with about 6 million refugees with PTSD and/or depression. Even if the current epidemiological research overestimates the prevalence of PTSD and depression in war survivors, it can be assumed that the absolute number of war survivors who do not migrate and suffer from PTSD and depression must still be many times higher than the number of refugees suffering from these disorders.

## Conclusions

The high number of war survivors with PTSD and/or depression has major implications for the societies concerned: (a) PTSD and depression can take a chronic course and lead to significant distress of the affected individuals as well as their partners and families; (b) the disorders are associated with functional impairments, affect wider interpersonal relationships, reduce productivity and generate substantial general health costs; and (c) research suggests a link between anger and PTSD (9), which can be the cause of further domestic violence, increase the desire for revenge (10) and thus increase the likelihood of future conflicts.

This large number is particularly concerning as the majority of war survivors live in areas with difficult political and socio-economic conditions. As such, unemployment, lack of opportunities for education, poor economic conditions—often in the form of extreme poverty—and a generally uncertain future may compound mental distress and complicate the recovery of people who suffer from mental distress. The existing evidence base suggests that there are effective psychological interventions to treat PTSD and comorbid depression in both adult (11) and young survivors of war (12) in LMICs. However, these treatments usually require a series of personal meetings with qualified mental health professionals, and in many countries there is neither the required funding for such costly treatments nor enough mental health professionals to implement them. To reduce the mental distress of war survivors with PTSD, new low-cost interventions are required that can be implemented on a large scale (13). These interventions may mobilize self-help potential in group-settings and involve modern technologies, given that access to the internet is widely and increasingly available even in most deprived world regions. Furthermore, there is a need for low-cost interventions that can be delivered by local providers within primary care settings in ways that are amenable to implementation within existing health structures and help to develop the long-term and sustainable capacity to reach a large number of survivors. However, although research developing such interventions is urgently required, this is not a priority for funding bodies that are usually situated in high-income countries, which are much less affected by war than LMICS and more concerned about refugees than about the people who still live in an area of former or ongoing armed conflict.

Considering the world-wide level of mental distress in war survivors that have not taken refuge, the attention of Western societies should focus not only on the refugees arriving at their shores but also on the much larger number of war-affected people who still live in their countries of origin, suffer from severe mental distress, are often exposed to adverse social conditions and do not have access to appropriate mental health care. Helping them might not only be regarded as a humanistic obligation, but also be an important political priority in the interest of global prosperity and peace.

## Author Contributions

NM wrote the first draft of the manuscript. All authors contributed to and have approved the final manuscript.

### Conflict of Interest

The authors declare that the research was conducted in the absence of any commercial or financial relationships that could be construed as a potential conflict of interest.
